# Die Europäische Union in der Corona-Krise

**DOI:** 10.1007/s10273-020-2667-6

**Published:** 2020-07-06

**Authors:** ZG Überblick

**Keywords:** H60, H77, H81

## Abstract

Die Corona-Krise hat alle Mitgliedstaaten der Europäischen Union getroffen — allerdings einige früher und härter als andere. Nun soll ein immens großer Rettungsschirm aufgespannt werden, über dessen Finanzierung, Konditionen und mögliche Auflagen die EU-Mitglieder noch streiten. Auch die Ebenen, auf der die Maßnahmen organisiert werden sollen, sind noch nicht ausbalanciert. Welche Bedeutung kommt dabei der nationalen, welche der supranationalen Ebene zu? Geht es vor allem um die Europäische Währungsunion oder um die gesamte EU? Solidarisches Verhalten gebietet nicht allein die europäische Idee, sondern auch die ökonomische Notwendigkeit, da die Mitgliedstaaten über Lieferketten stark miteinander verflochten sind.

## Abstract (en)

The Coronavirus crisis has affected al of thel member states in the European Union — but some earlier and harder than others. A large rescue package is to be set up, the financing, conditions and possible requirements of which are still being disputed by EU members. The levels at which the measures are to be organised have not yet been established either. What is the importance of the national level or the supranational leves? Is it primarily about the European Monetary Union or about the EU as a whole? Solidarity is not only required by the European idea, but also by economic necessity, since the member states are strongly connected by their supply chains.

